# Inflammatory Cytokines Changed in Patients With Depression Before and After Repetitive Transcranial Magnetic Stimulation Treatment

**DOI:** 10.3389/fpsyt.2022.925007

**Published:** 2022-06-02

**Authors:** Qiang Wang, Lingyun Zeng, Wenjuan Hong, Mingying Luo, Nan Zhao, Xiaofen Hu, Meili Shi, Jing Qiu, Yanmin Shen, Xiuju Teng, Haiying Min, Weiqing Liu

**Affiliations:** ^1^Clinical Research Center for Mental Disorders, Shanghai Pudong New Area Mental Health Center, School of Medicine, Tongji University, Shanghai, China; ^2^Department of Psychiatric Rehabilitation, Shenzhen Kangning Hospital, Shenzhen, China; ^3^Department of Anatomy, Histology and Embryology, Kunming Medical University, Kunming, China

**Keywords:** depression, repetitive transcranial magnetic stimulation, antidepressant effect, inflammatory cytokine, TNF-α, CRP-hc, IFN-γ

## Abstract

Studies have found that repetitive transcranial magnetic stimulation rTMS can produce antidepressant effects by affecting inflammatory cytokines in patients with depression, which plays a key role in the therapeutic mechanism of antidepressants. This study aimed to explore the changes in inflammatory cytokine levels in patients with depression after 4 weeks of rTMS treatment to determine the possible antidepressant mechanism of rTMS. This prospective, double-blind, pseudo-stimulus-controlled study was conducted, and a total of 57 patients with depression and 30 healthy controls were recruited. Patients were randomly divided into the active rTMS (*n* = 29) and sham rTMS groups (*n* = 28). The Hamilton Depression Scale was used to evaluate depressive symptoms and their severity. The serum levels of seven inflammatory cytokines were measured using enzyme-linked immunosorbent assay. Inflammatory cytokines include high-sensitivity C-reactive protein (CRP-hc); tumor necrosis factor (TNF-α); interferon (IFN-γ); interleukin-2 (IL-2); interleukin-4 (IL-4); interleukin-6 (IL-6); and interleukin-8 (IL-8). At baseline, TNF-α (*F* = 36.699, *p* < 0.001), IFN-γ (*F* = 8.907, *p* < 0.001), IL-4 (*F* = 66.256, *p* < 0.001), and IL-2 (*F* = 9.162, *p* < 0.001) levels in the depression group were significantly different from those of healthy controls. In the self-control analysis of the active rTMS group, the levels of IL-2 and CRP-hc increased significantly after 2 and 12 weeks of treatment. In the sham-rTMS group, IFN-γ increased after 2 and 12 weeks of treatment. Our results revealed that the changes in inflammatory cytokines after rTMS treatment showed different patterns compared to the sham group, suggesting that the antidepressant effect of rTMS may be related to changes in inflammatory cytokines.

## Introduction

Over the past two decades, there has been increasing evidence that depression is associated with a dysregulated immune system, including abnormalities in inflammatory markers (1). Compared with the healthy control (HC) group, the levels of pro-inflammatory cytokines and proteins in patients with acute phase depression were increased, and the results of the increase in interleukin (IL)-6, tumor necrosis factor (TNF)-α, and C-reactive protein (CRP) levels in the blood of patients with depression were relatively consistent (2). A recent meta-analysis of 82 studies, including 3,212 patients with depression and 2,798 HCs, showed that patients with depression had increased peripheral levels of IL-6, TNF-α, IL-10, soluble IL-2 receptor, chemokine ligand 2, IL-13, IL-18, IL-12, IL-1 receptor antagonist, and soluble TNF receptor 2, whereas interferon-gamma (IFN-γ) levels were decreased (3). In general, there is great heterogeneity in the data, which depends on the cytokine composition. This is at least in part due to the failure to consider the clinical course and course of disease, as well as the influence of potential confounding factors, such as comorbidity, drugs, fasting, smoking, and test methods (4–6).

Transcranial Magnetic Stimulation (TMS) is a non-invasive physical regulation method with antidepressant effects (7–10). It has been proven to have clinical application value for patients with depression (9, 11–13), including adolescents (14), the elderly (15, 16), patients with suicidal ideation (17) and patients with anxiety symptoms (18). These pulses can be transmitted at a high frequency (10–20 Hz) or low frequency (less than or equal to 1 Hz). Most clinical TMS treatments used to treat depression are usually performed at frequencies of 10–18 Hz (19, 20). In the early stages, there have been three large, multicenter, randomized pseudo-stimulus-controlled trials, including a total sample of 703 adult patients with major depression. One to four antidepressant treatments were ineffective in the enrolled patients. Two of these studies were industry-sponsored registered trials that led to Food and Drug Administration (FDA) approval of TMS treatment for depression in 2008 (10, 21). The evidence from all three trials was consistent, and TMS had statistically significant and clinically relevant benefits compared with pseudo-stimulation.

Few clinical studies have investigated the effect of rTMS on inflammatory cytokine levels in patients with depression. Langguth et al. (22) reported that peripheral levels of CRP and IL-6 increased after receiving 20-Hz rTMS in an elderly female patient with depression and rheumatoid arthritis, indicating that rTMS enhanced the patient’s peripheral inflammatory response. Zhao et al. (23) recruited 58 elderly patients with depression and 30 HCs. The peripheral levels of IL-1β and TNF were higher in the patient group than in the HC group at baseline. Compared with the non-rTMS treatment group, patients treated with 10-Hz rTMS for 4 weeks had IL-1β, and TNF in the peripheral blood levels decreased significantly.

However, the dynamic changes of inflammatory cytokine in depression before and after the TMS treatment are still largely unknown. The goal of this study was to explore the changes in inflammatory cytokine levels in drug-free patients with depression at recruitment and treated with antidepressants and 4 weeks of active/sham-rTMS treatment and to profile the inflammatory cytokine levels before (baseline) and after (week 2 and week 12) active/sham-rTMS treatment in patients with depression.

## Materials and Methods

### Sample and Study Design

The study participants were drawn from a consecutive clinical sample of adult patients, aged 18–80 years, who presented at the Shanghai Pudong New Area Mental Health Center, Tongji University School of Medicine. The hospital’s Ethical Committee approved the study and it was performed according to the Declaration of Helsinki. All participants provided written informed consent during the recruitment stage of the study. Patients with depression were diagnosed according to the Diagnostic and Statistical Manual of Mental Disorders, 5th Edition (DSM-5). The inclusion criteria were as follows: (1) age 18–80 years, (2) experienced an acute exacerbation of the symptoms of depression or had a baseline score of at least 14 points on the 24-item Hamilton Rating Scale for Depression (HAMD), (3) satisfied the DSM-5 criteria for depression based on the Structured Clinical Interview for DSM Disorders/Patient Edition, (4) having only one type of antidepressant during rTMS treatment and willing and capable of completing at least 20 sessions. None of the patients had a history of neurosurgery, seizures, head trauma, substance abuse or dependence, psychiatric or neurological disorders other than depression. Patients were excluded from the study if they have current inflammatory disorders, had received past rTMS treatment or other electromagnetic stimulations such as electroconvulsive therapy, ferromagnetic metallic implants, pacemakers.

We recruited 70 patients who were randomly and separately assigned to active (*n* = 36) or sham rTMS (*n* = 34) conditions. However, 13 patients (seven in the active-rTMS group and six in the sham-rTMS group) could not complete the study and withdrew their consent; thus, 57 patients (29 patients in the active-rTMS group and 28 patients in the sham-rTMS group) were finally included in the subsequent analysis. Independent, third-party assorted participants were enrolled into either the active or sham rTMS groups through computer-generated randomization numbers compiled through simple randomization. The clinical staff and patients were blinded to the assignment, except for one clinical technician who provided active or sham rTMS treatment according to the randomization numbers.

### Clinical Assessments

The primary outcome measure for the study was the score on the 24-item version of the HAMD (24). Seven factor scores including anxiety/somatization (items 10–13, 15, and 17), weight loss (item 16), cognitive disturbance (items 2, 3, 9, and 19–21), diurnal variation (item 18), retardation (items 1, 7, 8, and 14), sleep disturbance (items 4–6), and hopelessness (items 22–24) were calculated (25). Clinical assessment were administered at baseline (before rTMS treatment), mid-treatment (2 weeks), and 8 weeks after the last TMS session (12 weeks).

### Repetitive Transcranial Magnetic Stimulation

Patients were instructed to sit on a comfortable chair in a quiet room, and rTMS was delivered using a MagPro ×100 magnetic stimulator (Medtronic Co., Denmark) equipped with a butterfly coil. In the rTMS group, the stimulation was targeted to the position of the left unilateral dorsolateral prefrontal cortex, stimulated at a frequency of 10 Hz, power (intensity) level of 120% of the motor threshold, 80 trains, 30 pulses per train, 12-s intertrain-interval, 2,400 pulses per session, and five sessions per week. The same protocol was followed for the sham rTMS group using a fake coil that was unable to produce a magnetic field. The patients were blinded to the treatment received.

### Measurement of Inflammatory Cytokines

Venous blood samples were collected in anticoagulant-free tubes between 7 a.m. and 9 a.m. after an overnight fasting. The samples were kept at room temperature for 1 h, followed by centrifugation (3,000 g for 20 min at 4°C) for serum separation. Subsequently, the serum was separated and stored at –80°C until assayed. Serum levels of TNF-α, IFN-γ-, IL-4, IL-6, IL-8, IL-2, and CRP-hc for each sample were measured in duplicate using an enzyme-linked immunosorbent assay (ELISA) with a commercial human ELISA kit (Multisciences [Lianke] Biotech, Co., Ltd.), according to the manufacturer’s instructions. The concentrations of TNF-α, IFN-γ, IL-4, IL-6, IL-8, IL-2, and CRP-hc were expressed as picograms of protein per mL of serum. The intra- and inter-assay coefficients of variation were less than 9%.

### Data Analysis

**FIGURE 1:**
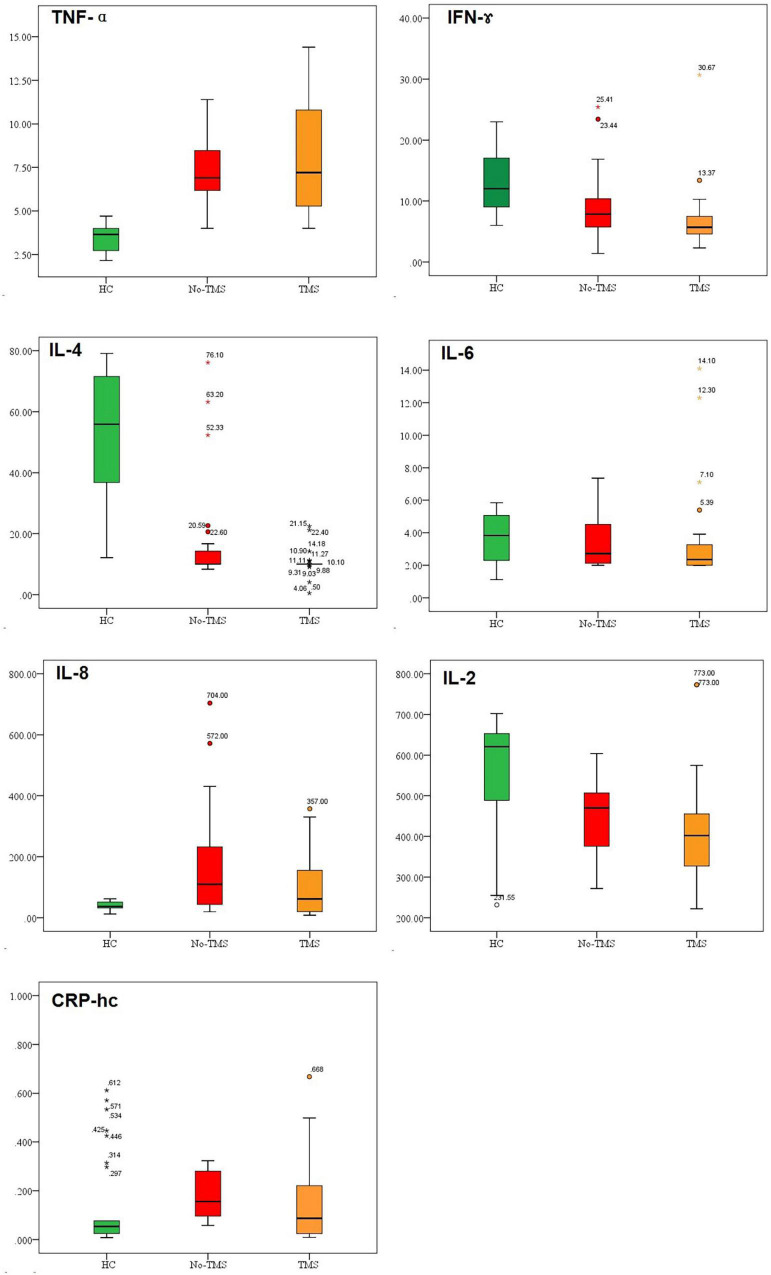
Baseline inflammatory cytokine levels in healthy controls (HC), active-rTMS (TMS), and sham-rTMS (no-TMS) groups in patients with depression.

The sociodemographic and clinical characteristics of our participants are presented as descriptive statistics, such as percentages and mean scores (standard deviation, SD) (Table 1). Analyses of variance (ANOVA) and χ^2^ tests were used to investigate differences in demographics, clinical variables, and antidepressant medications among the HC, active-rTMS, and sham-rTMS groups. The total and subscale HAMD scores before and after rTMS treatment were displayed and compared using an independent *t*-test (Table 2). Spearman correlation analysis was used to test the correlations between decreased HAMD scores and decreased cytokine levels. A box-plot diagram was created using GraphPad Prism software to analyze the differences in serum levels of TNF-α, IFN-γ, IL-4, IL-6, IL-8, IL-2, and CRP-hc at baseline in the active/sham-rTMS groups (Figure 1). The paired *t*-test was used for the self-controlled analysis of serum levels of inflammatory cytokines before and after treatment in the patient groups (Tables 3, 4).

**TABLE 1:** Demographic and clinical variables, comparison among healthy controls (HC), active-rTMS, and sham-rTMS groups.

Variables	HC	Active-rTMS	Sham-rTMS	Comparison	
				*F/t/*χ*^2^*	*p*
Cases (*n*)	28	29	28	–	–
Age [mean (SD)] years	56.5 (14.2)	58.2 (9.4)	55.6 (11.8)	*F* = 0.345	0.709
Age range (years)	29–76	38–72	29–77	–	–
Female [*n* (%)]	23 (82.1)	24 (82.8)	22 (78.6)	χ*^2^* = 0.189	0.910
Education [mean (SD)] years	7.3 (3.5)	8.0 (3.2)	7.4 (2.8)	*F* = 0.395	0.675
Single [*n* (%)]	1 (3.6)	3 (10.3)	3 (10.7)	χ*^2^* = 1.204	0.548
Married [*n* (*%*)]	24 (85.7)	21 (72.4)	21 (75.0)	χ*^2^* = 2.572	0.276
Divorce [*n* (*%*)]	3 (10.7)	3 (10.3)	2 (7.1)	χ*^2^* = 0.254	0.881
Widowed [*n* (*%*)]	0 (0)	2 (6.9)	2 (7.1)	χ*^2^* = 2.064	0.356
Family history [*n* (%)]	0 (0)	3 (10.3)	3 (10.7)	χ*^2^* = 3.174	0.205
Age of MD onset [mean (SD)] years	–	49.7 (13.7)	47.1 (14.8)	*t* = 0.639	0.491
Episode numbers [mean (SD)]	–	2.2 (1.3)	3.0 (2.1)	*t* = 1.705	0.094
Number of hospitalizations [mean (SD)]	–	1.6 (1.0)	2.1 (1.8)	*t* = 1.457	0.151
Course of MD [mean (SD)] months	–	9.1 (10.3)	8.0 (6.5)	*t* = 0.492	0.625
**Baseline HAMD [mean (SD)]**					
HAMD total score	–	28.5 (3.8)	33.1 (4.6)	*t* = 4.135	<0.001
HAMD-anxiety/somatization	–	8.0 (2.9)	9.0 (3.0)	*t* = 1.281	0.205
HAMD-weight	–	0.8 (0.8)	1.4 (0.8)	*t* = 2.744	0.008
HAMD-cognitive disturbance	–	3.1 (2.6)	4.0 (2.2)	*t* = 1.469	0.148
HAMD-retardation	–	2.0 (1.0)	2.2 (0.8)	*t* = 0.859	0.394
HAMD-diurnal variation	–	6.3 (1.5)	6.5 (1.6)	*t* = 0.457	0.650
HAMD-sleep disorder	–	4.9 (1.7)	4.9 (1.3)	*t* = 0.012	0.990
HAMD-desperation	–	4.3 (2.0)	4.0 (1.8)	*t* = 0.610	0.545
**Antidepressants (mg/day) [mean (SD)]**					
Duloxetine	–	40.0 (0)	40.0 (0)	–	–
Escitalopram	–	23.3 (3.9)	24.3 (4.3)	–	–
Paroxetine	–	50.0 (15.8)	44.0 (8.9)	–	–
Venlafaxine	–	206.3 (37.5)	178.1 (38.8)	–	–
Fluoxetine equivalent dose	–	54.9 (13.1)	51.7 (10.0)	*t* = 0.993	0.326

*HAMD, Hamilton Rating Scale for Depression; HC, healthy control. F/t/χ^2^, F, one-way ANOVA comparison among the three groups; t, independent sample t-test was used to compare between active-rTMS and sham-rTMS groups in patients with depression; χ^2^, kappa test.*

**TABLE 2:** Hamilton Rating Scale for depression (HAMD) scores at 2 and 12 weeks in the active-rTMS and sham-rTMS groups in patients with depression.

Variables	Active-rTMS		Sham-rTMS		Comparison	
	Mean	SD	Mean	SD	*t*	*p*
**Week 2**						
HAMD total score	14.41	2.970	17.14	2.772	–3.583	0.001
HAMD-anxiety/somatization	4.59	2.428	6.04	3.727	–1.733	0.090
HAMD-weight	0.24	0.577	0.54	0.693	–1.746	0.086
HAMD-cognitive disturbance	1.90	1.800	2.14	1.508	–0.559	0.578
HAMD-retardation	0.72	0.702	0.86	0.803	–0.666	0.508
HAMD-diurnal variation	3.14	1.356	3.21	1.371	–0.211	0.833
HAMD-sleep disorder	2.03	1.569	2.46	1.575	–1.032	0.307
HAMD-desperation	1.79	1.521	1.89	1.133	–0.280	0.781
**Week 12**						
HAMD total score	7.90	2.257	6.54	2.317	2.246	0.029
HAMD-anxiety/somatization	3.62	2.243	2.75	1.602	1.681	0.098
HAMD-weight	0.07	0.258	0.04	0.189	0.554	0.582
HAMD-cognitive disturbance	0.83	0.848	0.79	0.957	0.175	0.862
HAMD-retardation	0.45	0.572	0.43	0.573	0.130	0.897
HAMD-Diurnal variation	1.66	0.897	1.46	1.036	0.744	0.460
HAMD-sleep disorder	0.97	1.017	0.75	0.799	0.887	0.379
HAMD-desperation	0.31	0.541	0.32	0.670	–0.069	0.945

*HAMD, Hamilton Rating Scale for Depression. t, independent sample t-test was used to compare active-rTMS and sham-rTMS groups in patients with depression.*

**TABLE 3:** The levels of inflammatory cytokines at baseline, 2 and 12 weeks in the active-rTMS and sham-rTMS groups in patients with depression.

Variables		Baseline		Week 2		Week 12	
		Mean	SD	Mean	SD	Mean	SD
Active-rTMS	TNF-α	8.106	3.202	9.905	7.457	6.980	2.883
	IFN-γ	7.014	5.164	6.752	2.151	5.733	2.013
	IL-4	10.479	3.871	15.648	39.190	8.318	4.441
	IL-6	3.486	2.928	3.960	2.360	3.813	1.657
	IL-8	202.341	541.459	338.238	276.709	260.658	274.033
	IL-2	410.345	130.926	470.586	150.389	432.069	173.358
	CRP-hc	0.139	0.192	0.299	0.312	0.366	0.253
Sham-rTMS	TNF-α	7.333	1.860	8.291	2.856	6.580	2.507
	IFN-γ	9.172	5.562	6.248	1.998	5.500	2.140
	IL-4	17.169	17.126	12.060	17.279	7.171	3.596
	IL-6	3.375	1.591	4.339	4.332	4.792	3.423
	IL-8	175.739	179.681	514.150	1097.508	275.171	233.763
	IL-2	448.536	93.633	475.464	117.294	508.429	151.898
	CRP-hc	0.271	0.701	0.193	0.161	0.428	0.400

**TABLE 4:** Self-controlled comparisons for serum levels of inflammatory cytokines before and after treatment in patients with depression.

Variables		Baseline vs. week 2		Baseline vs. week 12		Week 2 vs. week 12	
		*t*	*p*	*t*	*p*	*t*	*p*
Active-rTMS	TNF-α	–1.148	0.261	1.745	0.092	2.109	0.044
	IFN-γ	0.300	0.767	1.266	0.216	2.171	0.039
	IL-4	–0.705	0.487	1.790	0.084	1.018	0.317
	IL-6	–0.897	0.377	–0.521	0.606	0.274	0.786
	IL-8	–1.093	0.284	–0.492	0.627	1.262	0.217
	IL-2	–2.278	0.031	–0.557	0.582	1.068	0.294
	CRP-hc	–2.448	0.021	–3.681	0.001	–0.874	0.390
Sham-rTMS	TNF-α	–1.562	0.130	1.201	0.24	2.456	0.021
	IFN-γ	2.602	0.015	3.402	0.002	1.474	0.152
	IL-4	1.070	0.294	3.084	0.005	1.409	0.170
	IL-6	–1.087	0.287	–1.872	0.072	–0.406	0.688
	IL-8	–1.604	0.120	–1.725	0.096	1.138	0.265
	IL-2	–0.941	0.355	–1.774	0.087	–1.043	0.306
	CRP-hc	0.567	0.575	–1.151	0.26	–3.346	0.002

*The paired t-test was used for self-controlled analysis of serum levels of inflammatory cytokines pre- and post-treatment in patients in the active and sham rTMS groups. The level of statistical significance was set at a two-tailed P value of 0.05.*

## Results

### Demographic and Clinical Characteristics

As shown in Table 1, there were no significant differences in baseline age, years of education, marital status, or family history among the active-rTMS, sham-rTMS, and HC groups. Compared with the sham rTMS group, the HAMD total score in the active-rTMS group was significantly lower than that in the pseudo-stimulation group. The main difference in each HAMD factor score is the body weight factor. There was no significant difference in the antidepressant dose between the active-rTMS and sham-rTMS groups. Table 1 lists the demographic and clinical characteristics and antidepressant exposure of the three groups.

### Clinical Characteristics Before and After Treatment

As shown in Table 2, the HAMD total score at 2 weeks between the active-rTMS and sham-rTMS groups was statistically different. The total score on the 2-week HAMD in the active-rTMS group was significantly lower than that in the sham-rTMS group. At 12 weeks, the total HAMD score in the active rTMS group was significantly higher than that in the sham rTMS group.

### Baseline Inflammatory Cytokines

As shown in Figure 1, the serum levels of TNF-α, IFN-γ, IL-4, and IL-2 at baseline in the HC, active rTMS, and sham rTMS groups were significantly different. The difference mainly lies in the fact that the baseline TNF-α level was significantly lower in the HC group than in the patients with depression in either the active-rTMS or sham-rTMS groups. The level of IFN-γ, IL-4, and IL-2 were significantly higher in HC group than that of the depression group.

### Changes of Inflammatory Cytokines Before and After Treatment

The levels of several inflammatory cytokines in the active-rTMS and sham-rTMS groups changed significantly after 2 and 12 weeks of treatment (Table 3). In the self-control analysis of active-rTMS group, the levels of IL-2 and CRP-hc changed significantly after 2 weeks of treatment, the level of CRP-hc changed significantly after 12 weeks of treatment, and the level of TNF-α and IFN-γ changed significantly between week 2 and week 12. In the self-control analysis of sham-rTMS group, IFN-γ changed significantly after 12 weeks of treatment. The level of IFN-γ and IL-4 changed significantly, and the level of TNF-α and CRP-hc changed significantly between week 2 and week 12 (Table 4).

### Correlation Between Decreased Depressive Symptoms and Inflammatory Cytokines After Transcranial Magnetic Stimulation Treatment

After the active or sham TMS treatment, the decrease in depressive symptoms of the patients (*n* = 57) were negatively correlated with the decreased levels of TNF- α after 2 weeks of treatment. While the decrease in depressive symptoms were negatively correlated with the decreased levels of IL-2 at 12 weeks. The changes of all the other inflammatory cytosines were not significantly correlated with the decreased depressive symptoms in the patients (Table 5).

**TABLE 5:** Correlations between decreased HAMD score and decreased serum levels of inflammatory cytokines before and after TMS treatment in patients with depression.

Inflammatory cytokines	Baseline-week 2 Δ HAMD		Baseline - week 12 Δ HAMD	
	*r*	*p*	*r*	*p*
ΔTNF-α	–0.272	0.040	–0.089	0.511
ΔIFN-γ	–0.117	0.387	–0.143	0.289
ΔIL-4	0.134	0.320	0.280	0.035
ΔIL-6	–0.022	0.869	–0.081	0.551
ΔIL-8	–0.029	0.831	–0.008	0.955
ΔIL-2	–0.103	0.446	–0.340	0.010
ΔCRP-hc	0.019	0.886	–0.163	0.226

## Discussion

### Main Findings

In this study, we hypothesized that, in association with depressive symptom improvements, serum inflammatory cytokine levels in patients with depression would be changed after active rTMS treatment compared with the sham rTMS group. The main findings supported the assumption that the change patterns of inflammatory cytokine levels highly varied after rTMS treatment. In the active rTMS group, the serum levels of CRP-hc and IL-2 significantly changed at week 2, and the change last through the 12 weeks period, which were not seen in the sham-rTMS group, suggesting that rTMS can change serum inflammatory cytokine levels in patients with depression. Furthermore, the improvement of depressive symptoms in the patients were negatively correlated with the decreased levels of TNF-α during the TMS treatment at 2 weeks and negatively correlated with decreased IL-2 levels at 12 weeks after the treatment. Besides, the baseline TNF-α level was significantly higher and IFN-γ, IL-4, and IL-2 were lower in patients with depression compared with the HC group. After the to the best of our knowledge, this is the first study to examine dynamic changes in the levels of inflammatory cytokines following active rTMS and sham-rTMS in patients with depression.

### TNF-α and Depression

This study found that the concentration of TNF-α in the depression group was significantly higher than that in the HC group, but there was no significant change after 2 and 12 weeks of antidepressant plus active or sham rTMS treatment. However, serum TNF-α levels in the active TMS group was significantly decreased from weeks 2 to weeks 12, indicating a dynamic effect of TMS on TNF-α levels at different treatment period. Interestingly, the early improvement of depressive symptoms among the patients was negatively correlated with the decrease of TNF-α levels, suggesting that early stage TMS treatment may have adverse effects of increasing TNF-α levels. At present, it is generally believed that TNF-α is an important pro-inflammatory cytokine, which has attracted much attention because it can cause inflammatory and apoptotic cell death and mediate the release of a variety of cytokines (26). Two mechanisms may link TNF-α to the pathophysiology of depression. First, TNF-α may regulate the activity of neuronal serotonin transporters and stimulate serotonin uptake (27). Second, TNF-α activates tryptophan- and serotonin-degrading enzyme indoleamine-2,3-dioxygenase, resulting in reduced availability of serotonin in patients with depression (28). From the perspective of the mechanism, it has been assumed that the increased production of TNF-α may play a pathogenic role in depression, and the TNF-α levels of patients with depression vary greatly in many clinical studies. TNF-α levels have been reported to be elevated in patients with depression (29–31), unchanged (32), and decreased (33). One possible explanation for this inconsistency is that different TNF-α expression patterns may occur in different clinical subtypes of depressive episodes (34, 35).

### High-Sensitivity C-Reactive Protein and Depression

We found significant increases in CRP-hc levels after 2 weeks of TMS treatment and last through the whole 12 weeks of follow-up in the active-rTMS groups. CRP is a non-specific acute-phase protein, and its level increases in response to an inflammatory response. A meta-analysis of cross-sectional studies confirmed that the average concentrations of CRP in patients with depressive episodes were higher than those in the control group (36). Cheng et al. reported that in patients treated with antidepressants, baseline CRP levels were significantly correlated with treatment response at 2 weeks. Higher CRP levels were associated with poorer treatment outcomes. After 6 weeks, CRP levels in both treatment groups increased significantly (37). It is speculated that CRP may serve as a useful marker of inflammation in the antidepressant treatment of depression (38).

### IFN-γ and Depression

In this study, IFN-γ decreased after 2 and 12 weeks of antidepressant treatment with sham-rTMS and decreased from 2 to 12 weeks in the active-rTMS group. Chen et al. (39) found that the baseline IFN-γ levels in patients with depression were significantly higher than those in the control group. In the antidepressant treatment group, a decrease in IFN-γ levels was observed after 8 weeks of treatment. The decrease in IFN-γ level was not statistically significant in the remission group, while the decrease in IFN-γ levels was statistically significant in the non-remission group. Dahl et al. (40) found that, compared with HC, the IFN-γ level of untreated patients with depression was significantly higher. In the group of patients who achieved remission, IFN-γ levels decreased significantly compared to baseline levels. These results suggest that IFN-γ may be involved in the pathogenesis of depression and in the therapeutic mechanism of antidepressant treatment.

### Inflammatory Cytokines and Repetitive Transcranial Magnetic Stimulation

This study found that rTMS had an effect on various inflammatory factors during antidepressant treatment, which was significantly different from that in the sham-rTMS group. An interesting study by Zhao et al. (23) reported the effects of rTMS on IL-1β and TNF-α levels in elderly patients with refractory depression. In their study, serum levels of IL-1β and TNF-α were measured before the study, 48 h, and 1, 2, 3, and 4 weeks after the first TMS treatment. The levels of IL-1β and TNF-α gradually decreased and were significantly lower than those in the control group. Serum factors of healthy individuals were not affected by rTMS. The levels of IL-1β and TNF-α were positively correlated with the HAMD score. These results suggested that rTMS may reduce serum IL-1β and TNF-α levels during antidepressant treatment. Interestingly, our results showed that IL-2 levels in the depression patients was significantly decreased at the baseline and increased after 2 weeks of active TMS treatment. Furthermore, the long-term improvement of depressive symptoms in the patients was significantly correlated with the change of IL-2 levels, in supporting of the pathological role of IL-2 in the development and TMS treatment of depression.

### Limitations

The present study had a few limitations. First, the sample size of the diagnostic subgroup was small. The number of patients in each group was smaller after stratification according to active rTMS and sham rTMS. Another limitation is the uncertainty over how accurately serum inflammatory cytokine levels reflect levels in the brain. Third, due to ethical reasons, all the depressive patients in this study were administered by antidepressants besides the TMS or sham-TMS treatments, so the effects of antidepressants on serum cytosines could not be excluded. The current study relied only on the assessment of depressive symptoms, which is inadequate.

## Conclusion

Despite these limitations, we performed a careful evaluation of depressive symptoms, as well as dynamic measuring of the serum inflammatory cytokines throughout the active/sham-rTMS treatment period, thus obtaining reliable results. This study preliminarily explored the characteristics of inflammatory cytokines in patients with depression and assessed the dynamic changes of these cytokines 2 and 12 weeks after active/sham-rTMS treatment. Our results revealed that there were significant changes in several inflammatory cytokines in patients with depression compared with HC, and the changes in inflammatory cytokines in the rTMS treatment group followed different patterns compared with the sham control group, suggesting that the antidepressant effect of rTMS is related to the changes in inflammatory cytokines.

## Data Availability Statement

The raw data supporting the conclusions of this article will be made available by the authors, without undue reservation.

## Ethics Statement

The studies involving human participants were reviewed and approved by the Ethical Committee of Shanghai Pudong New Area Mental Health Center. The patients/participants provided their written informed consent to participate in this study.

## Author Contributions

QW designed the study and drafted the manuscript. LZ conceived the analysis. WH, ML, NZ, and XH performed the analysis and interpretation of the results. MS, JQ, YS, and XT collected the data. HM and WL supervised the experiment and reviewed the manuscript. All authors contributed to the article and approved the submitted version.

## Conflict of Interest

The authors declare that the research was conducted in the absence of any commercial or financial relationships that could be construed as a potential conflict of interest.

## Publisher’s Note

All claims expressed in this article are solely those of the authors and do not necessarily represent those of their affiliated organizations, or those of the publisher, the editors and the reviewers. Any product that may be evaluated in this article, or claim that may be made by its manufacturer, is not guaranteed or endorsed by the publisher.
